# Is the digitalisation the future of the luxury industry?

**DOI:** 10.1016/j.heliyon.2024.e40029

**Published:** 2024-11-01

**Authors:** Francisco Sanz-Lopez, Rocío Gallego-Losada, Antonio Montero-Navarro, Elisa García-Abajo

**Affiliations:** a Department of Financial Economy and Accountancy, Facultad de Ciencias de la Economía y de la Empresa, Universidad Rey Juan Carlos, Paseo de los Artilleros, s/n, 28032, Madrid, Spain; b Department of Business Economics (Adm., Dir. and Org.), Applied Economics II and Fundamentals of Economic Analysis, Facultad de Ciencias de la Economía y de la Empresa, Universidad Rey Juan Carlos, Paseo de los Artilleros, s/n, 28032, Madrid, Spain

**Keywords:** Luxury, Prestige brand, Digital transformation, Artificial intelligence, E-Commerce

## Abstract

In the context of the fourth industrial revolution, this study examines the digital transformation of the luxury industry through a bibliometric analysis of 236 articles (1991–2022) and a bibliographic coupling of research from 2022 to 2024. The analysis identifies key trends, including the use of digital tools to enhance customer engagement, the growing role of review-based feedback in the tourism sector, value creation through innovative products and promotional strategies, and the advances in sustainability via digitalization. Despite these hot trends, many firms in the luxury sector have not yet involved in a real digital transformation, as they have only digitalized specific aspects of their operations. The findings suggest that while luxury firms are embracing digital tools to meet the expectations of digitally native consumers, they face the challenge of balancing innovation with the preservation of their core values. The study concludes that digital transformation is not merely an operational shift but a strategic imperative for the future competitiveness and profitability of the luxury sector.

## 1 Introduction

The global luxury sector has witnessed a substantial growth in the most recent years, with a total market turnover of €1.5 trillion in 2023 [1]. In 2022, the top 100 luxury goods companies generated $347 billion in sales [2], with all major brands reporting positive growth [3], confirming the sector's economic relevance [4].

The term luxury means comfort, exclusivity, superior craftsmanship and personalization. The brands and houses operating in this sector show a special sense of uniqueness, reputation, and innovation [[5], [6], [7]]. According to Veblen [8], luxury is manifested through conspicuous consumption, which refers to spending money on goods and services that are not necessary for life or comfort but are intended to display a person's or group's social status and economic power. The upper classes use this type of consumption to demonstrate their wealth and social standing, where status is measured by the ability to spend on items without practical function [9].

The luxury market covers a broad spectrum of industries, from the automotive sector to fashion. Products in this industry must possess certain common characteristics, with uniqueness and quality being paramount [10]. A luxury product can be defined as a work of art designed for an exclusive market. Generally, luxury brands exhibit a low functionality-to-price ratio but a high intangible utility and price. Shared attributes of these products include craftsmanship, design, exclusivity, and reputation [11].

Luxury goods can come in a wide range of prices, referred to as "accessible luxury," which allows luxury brands to offer products at a lower price, accessible to consumers who previously did not buy luxury goods [12]. The consumption of luxury goods is associated with the success of consumers, revealing their status. Three types of consumers can be identified: uniqueness seekers, compulsive shoppers, and public interest-oriented consumers [13].

Luxury goods and services can be classified into five categories. First, tangible goods include fashion, accessories, automobiles, jewellery, high-end technology, and fine art. Second, luxury services encompass hotels, fine dining, exclusive travel, and wellness experiences. Third, luxury real estate covers high-end homes and private vacation properties. Fourth, luxury financial services like private banking and bespoke insurance, tailored to affluent clients. Finally, personalised services and exclusive experiences cater to individual preferences, offering unique and prestigious consumption [14,15].

Luxury products can be divided into bags and accessories, beauty products, clothing and footwear, jewellery and watches, and multiple luxury goods [2]. In addition, various studies offer more detailed classifications. Jee Han et al. [16] highlight three main types of luxury products: designer handbags (from brands like Louis Vuitton and Gucci), luxury shoes for men and women, and luxury automobiles. Wang and Griskevicius [17] further expand this classification into five categories: designer handbags, designer shoes, luxury jewelry (used as status signals), luxury watches (as symbols of prestige), and luxury automobiles. Tynan et al. [18] define luxury products as high-quality, expensive, non-essential, exclusive, prestigious, and authentic goods. This definition reinforces the idea that luxury products are not only functional but also convey status and exclusivity. Finally, following the value-based segmentation approach of Wiedmann et al. [19], luxury consumption behaviour is driven by factors such as exclusivity, superior quality, and authenticity, which distinguish luxury goods from conventional products.

Luxury fashion brands like Chanel, Louis Vuitton, Gucci, and Hermès leverage innovation and tradition to maintain their prestigious status. These brands emphasize superior craftsmanship, unique design, and exclusivity [20]. To engage younger consumers, they use fashion films to showcase their heritage and values [21]. Contemporary luxury fashion shows exhibit hybridity, blending time, space, cultures, arts, nature, and technology to express brand identity and philosophy. For instance, Chanel represents modern elegance, while Gucci aligns with subculture [22].

In luxury hospitality, brands like Ritz-Carlton and Four Seasons focus on exceptional service and personalised experiences, with customer satisfaction and service quality as key factors [23]. Research suggests that empowering employees to make decisions that ensure guest satisfaction is essential for achieving service excellence [24]. Similarly, haute cuisine restaurants offer multisensory experiences, combining quality ingredients with carefully designed settings [25,26]. The presentation of dishes, interaction with chefs, and timing of service significantly impact diners' emotional responses and perceptions [27]. These restaurants have recently embraced digitalization to cater to status-oriented consumers seeking exclusivity [28].

The implementation of new technologies will increase sales by €120 billion in 2030 for companies in the luxury sector [29]. The opportunities offered by disruptive technologies mean that companies are now operating in an increasingly competitive environment, with a trend toward globalisation supported by information and communication technologies (ICTs). In this environment, firms in the luxury sector face diverse threats but can take advantage of opportunities arising from the digital transformation of society itself. Within this traditional sector characterised by a certain resistance to modernity, such changes are nonetheless being introduced and are the object of this study. Digitalization has introduced new tools such as chatbots and social media platforms, offering personalised customer interactions and reshaping company structures [[30], [31], [32], [33], [34]]. The options provided by ICTs thus make it possible to offer a customer complementary service that meet the standards of the luxury shopping experience [30].

More specifically, the digitalization of the luxury sector has led to a number of positive developments, such as the ability to implement online pricing strategies [35]; adaptation to consumer behaviours, particularly those of Generation Z [36]; the use of review platforms to understand the most valued attributes in products or services [37]; improving brand image projection through social media [38]; verifying the authenticity of luxury items by determining their provenance [39]; and the creation of new digital luxury products. Although scarce, these digital products generate high interest among customers seeking status-driven consumption [40].

The digital revolution has sparked new product innovations [41], leading to an increased interest in the luxury sector and expanding beyond premium offerings [42]. Although initially reluctant to digital transformation [43], companies in the luxury sector reached a turning point during the Covid-19 pandemic [44], when they had to confront the digital environment to maintain their positions; and in the two years following the pandemic, the metaverse was pioneered [45]. The explosion of ICTs has caused a unification of the physical and digital worlds, impacted all sectors of the economy and gave rise to the so-called fourth industrial revolution [46]. Companies that fail to introduce technological innovation into their organisation run the risk of significant competitive disadvantage [47].

Given the paramount importance of digital transformation, especially within the luxury sector, where the challenge of preserving exclusivity adds layers of complexity, this study endeavours to delineate the foremost research trends.

To achieve its objective, this study uses a bibliometric analysis, which identifies the evolution of a scientific field and its emerging areas. Several scientific articles published in English in peer-reviewed journals from the Web of Science (WoS) Core Collection were selected, excluding book chapters, working papers, communications, and conference proceedings. The selection was based on keyword searches in titles, abstracts, and author keywords, covering the period from January 01, 1991 to January 01, 2023. The initial selection of 353 articles was refined through verification and the exclusion of irrelevant papers, resulting in a final sample of 236 articles. Scientific productivity was analysed by assessing the number of publications, and the distribution of articles by country, institution, journal, and author, as well as the most cited articles. In addition, relational techniques were used to analyse the structure of the research topic through scientific mapping, based on co-word analysis, co-citation analysis and bibliographic coupling to identify the future trends.

The study offers three main contributions: first, it demonstrates how digitalization is reshaping the luxury sector by enabling companies to maintain their exclusivity while adopting new technologies; second, it identifies the core research areas as the impact of digitalization, the application of digital tools, and new communication channels through social media; third, it underscores the necessity for luxury brands to balance digital innovation with their core values of heritage and exclusivity. These findings suggest that while the luxury sector has been cautious in its digital transformation, the COVID-19 pandemic has accelerated this process, leading to innovative practices and new market opportunities.

According to this objective, our study is comprised of 5 sections. Following this introduction, Section 2 reviews the background literature on the luxury sector and poses the research questions. Section 3 explains the methodology used in the study. Section 4 presents the results and analyses then. Finally, section 5 includes the conclusions and examines the outstanding gaps in the existing body of literature. Therefore, this study could serve as a basis for future research and identify the main forms of digitalization.

## 2 Background and research questions

The lack of studies on digitalization in the luxury sector, covering all industries, justifies this paper. Digital transformation can be approached from two distinct perspectives [47,48]; in terms of the use of digital technologies, it can improve the performance of company processes [49]; from a more far-reaching perspective, it means a broad transformation of the business model [50]. In this way, digitalization refers to converting analogue information to digital formats, while digital transformation implies deeper changes in business models, products, and processes [50,51]. In this sense already in 2009, Okonkowo [42] identified that luxury companies should not only recognize the need to adopt new platforms but also reconsider all aspects of their business. In the many studies examined, digital adaptation was done in these two ways.

Digitalization in the luxury sector encompasses numerous topics, including: the use of social media [34,52]; the art of storytelling [53]; communications [54]; digital marketing [55]; digitalization [32]; online sales [56]; company websites [57]; and the use of digital platforms by luxury hotels, along with the impact of online reviews [58]. Despite the importance of digital transformation in organisations and recognition of the need [59], comprehensive examinations of the digital transformation have appeared in the academic literature for only a few companies in the sector, as in the case of Burberry [60]. At this point, ongoing scientific development around the study of digitalization and digital transformation in luxury companies highlights the need for specific analysis of the intellectual structure of this area of knowledge.

Following Zupic and Cater [61], “Synthesizing past research findings is one of the most important tasks for advancing a particular line of research” (p. 429). Donthu et al. state that [62], “scholars use bibliometric analysis for a variety of reasons, such as to uncover emerging trends in article and journal performance, collaboration patterns, and research constituents, and to explore the intellectual structure of a specific domain in the extant literature” (p. 285). The same authors remark that the volume of data used in such kind of approach, a bibliometric analysis, includes hundreds or even thousands of articles analysed, being the most adequate technique when dealing with massive data.

Eight scientific literature reviews in the field of luxury companies have been identified, some of which are bibliometric studies (Table 1).

**Table 1:** Literature review, articles on luxury goods and services.

Reference	Methodology	Data base	Time	Objective	Conclusion
[63]	Bibliometric	Web of Science (WoS), Scopus and China National Knowledge Infrastucture	Before 2021	Identification of research trends comparing the value creation practices carried out within the Chinese tourism sector with those conducted in other countries.	Thirteen thematic areas were stablished in which both similarities and differences were identified between the research conducted in China and those performed in other regions.
[64]	Bibliometric	Scopus	Before 2019	Comprehensive overview of the works published in the scientific literature on the luxury sector encompassed within the area of international marketing.	Six main topics were determined: social media, artificial intelligence, sustainability, new forms of luxury and experiences.
[65]	Bibliometric	Scopus	1996–2019	Presentation of the current research lines in the field of luxury brands.	Seven basic categories are shown in the study: Luxury value, ostentatious consumption, value creation, perception, brand value, and counterfeits.
[66]	Bibliometric	Scopus	1982–2019	Evaluation of the impact of artificial intelligence on branding strategies and its contribution to improving business performance.	Eight predominant research trends are outlined, highlighting the role of artificial intelligence in influencing sales, social networking and pricing strategies, and emphasizing AI as a critical area for both practical and theoretical exploration.
[67]	Bibliometric	Scopus	1984–2020	Exposition of the research trends followed by publications in Psychology and Marketing highlighting new, emerging and promising research topics.	Future research areas are suggested including luxury consumption behavior, consumption ethics, value assessment, and the impact of social desirability.
[68]	Bibliometric	WoS	1985–2019 (August)	Analysis of the research on sustainable luxury (SL) and identifying future research lines.	Eight clusters were delimited to group the research papers: Production and supply chain management, sustainability, consumer, advanced technology, marketing, organization, human resource management and business law.
[69]	Bibliometric	WoS	2000–2015	Analysis of the current state of the art on luxury and future research proposals.	The scope of research covers diverse fields, showing a transition from sociological research to management studies, with a current emphasis on applied research.
[70]	Bibliometric	Wos	1993–2022	Identification and categorization of the main research streams in the field of luxury.	Four main areas of research have been defined in the analysis: Contribution of luxury tourism to development; Luxury shopping in tourism; Demand behavior in luxury tourism, and Digital Transformation in luxury tourism.

Table 1 shows the eight bibliometric articles related to the luxury sector prior to this research, as well as the databases used in each one of them, the time frame covered by the research, and their main objective and conclusion. The main limitations found in these studies are then analysed, justifying the need for a bibliometric analysis focused on the digitalization of the luxury sector.

Three of the papers listed in Table 1 are only indirectly related to the luxury sector. Liu et al. [63] conduct a holistic review of articles on value creation in tourism using a bibliometric methodology. This article focuses on the hotel sector in China, and although the paper is not exclusively dedicated to the luxury market, the authors implicitly refer to the market by including the aspect of ‘the luxury accommodation experience’. Donthu et al. [67] offer a bibliometric study that analyses the impact of psychology in the marketing sector, addressing the topic of ‘luxury’ in only one of the eight groups under study. Varsha et al. [66] conduct a bibliometric analysis of the impact of artificial intelligence (AI) on branding. This work does not focus on luxury brands, but these brands do appear as one of nine groups in the analysis of co-occurrence.

Two of the papers in Table 1 take the luxury sector as their main focus. Husain et al. [65] identify research trends in luxury branding through a systematic literature review, not focusing on a specific topic but analysing all Scopus-listed publications from 1996 to 2020, regarding luxury brands. Gurzki and Woisetschlaeger [69] provide a review of the state of research in the luxury sector, selecting the topic from all disciplines and identifying ten main research streams, without specialising in digitalization.

The remaining three papers, focus on luxury combined with another item. Veloutsou et al. [64] analyse international luxury marketing, while Shashi et al. [68] conduct a bibliometric analysis on sustainable luxury, aimed at developing a suitable system in the supply chains of luxury companies that base their strategies on sustainability and the circular economy. Lopes et al. [70] focus their research on luxury tourism, identifying four groups, including one that analyses digital transformations in luxury tourism.

Thus, only two of the mentioned works share some aspects with this study – i.e., those studies that are more directly related to digitalization and digital transformation in the luxury sector. Specifically, Varsha et al. [66] analyse the generation of brand value through AI, focusing on the application of a single tool rather than digitalization and digital transformation in a broad sense. Moreover, their focus is not exclusively on the luxury sector but on any type of brand. Likewise, the work of Veloutsou et al. [64] does not analyse the sector's digital transformation in a broad sense but focuses on international marketing in the luxury sector, with any research not related to novel forms of marketing removed from consideration. Therefore, there is a lack of comprehensive bibliometric studies on digitalization in the luxury sector, revealing a clear need for further analysis.

According to Demirkan et al. [71], digital transformation (DT) is “the profound and accelerating transformation of business activities, processes, competencies, and models to fully leverage the changes and opportunities brought by digital technologies and their impact across society in a strategic and prioritized way” (p. 14). Digital transformation leads to what has been called the Fourth Industrial Revolution [72], entirely reshaping business models and sectors [73] including, as this paper shows, the luxury industry.

Therefore, it is essential to carry out a comprehensive and systematic approach to the digital transformation of the luxury sector, covering all its constituent elements, including products, services, tools, and the sector as a whole. This will become the main contribution of this article, which is to provide an overview and synthesis of the current research on the notion and practices of digitalization and digital transformation in the luxury sector.

This general objective can be broken into the following specific aims:

1. Assess academic productivity by analysing the historical evolution of publications.
2. Determine the intellectual structure of the research topic.
3. Discover the thematic organisation of the research topic.
4. Identify the conceptual structure of the research topic.
5. Analyse specific research trends on digitalization in the luxury sector.

According with these objectives, and in light of recent bibliometric reviews, the following research questions are posed:

• RQ1. Which are the main phases of the academic literature on digitalization in the luxury industry?
• RQ2. Which are the academic groundings of the intellectual structure of the topic?
• RQ3. Which are the main academic schools that gather the research dealing with the digitalization in the luxury sector?
• RQ4. Which are the main subthemes gathering the attention of the academic community?
• RQ5. Which is the most probable research agenda in this topic?

In sum, this paper offers to readers a holistic overview of the impact of digitalization on a specific sector of the luxury, which is relevant due to the circumstances of this exclusive sector.

## 3 Methodology

**Fig. 1:**
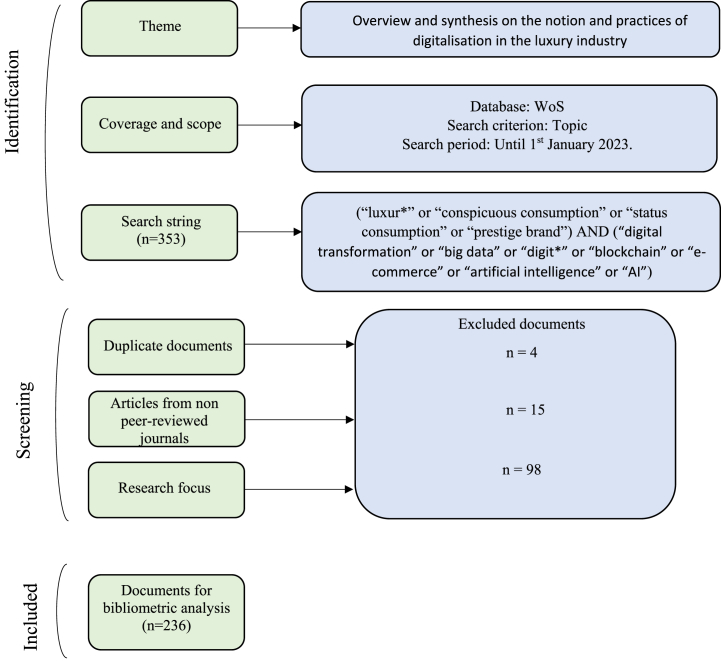
Selection process using PRISMA methodology.

**Source:** Own elaboration.

In order to fulfil the objectives of this work, a bibliometric methodological approach has been adopted, making it possible to identify the evolution of a scientific field together with its emerging areas [62,74]. A selection process was carried out using the PRISMA methodology (Fig. 1).

To begin this bibliometric review, a selection of papers was made based on a keyword search of the literature, excluding possible researcher bias. The selected papers include only scientific articles published in peer-reviewed journals in English language included in the Web of Science Core Collection, it is commonly used as a source of data for scientific and academic research [75], excluding from the analysis book chapters, working papers, communications, and conferences. The search criteria employed encompassed all entries with any permutation of the terms in their title, abstract and author keywords (Topic). The search has been intentionally restricted to metadata, as searching the terms throughout the texts would result in a relatively high number of false positives [76], as many articles might reference digitalization or luxury, without them being the focus of their research. The final keywords used constitute the following logical conjunction:

1. “luxur∗” or “conspicuous consumption” or “status consumption” or “prestige brand” (Topic)
2. AND “digital transformation” or “big data” or “digit∗” or “blockchain” or “e-commerce” or “artificial intelligence” or “AI”.

The keywords were chosen based on their relevance and frequency in the literature, ensuring a comprehensive capture of the field's scope. Luxur∗ is the central keyword, while conspicuous consumption and status consumption are terms derived from one of the main studies of the term luxury [8,9]. Prestige brand, in turn, is frequently used in the academic literature referring to companies that market luxury products [77]. On the other hand, dealing with the digital transformation of the industry, the central keyword was digit∗, which encompasses both digitalization and digital transformation. Blockchain, e-commerce, big data and AI were also included, as they are some of the most frequently used tools in the digitalization/digital transformation of a company [78,79].

The search covered the period from 1/01/1991 to January 01, 2023. This first selection resulted in a total of 353 articles. All results obtained were double-checked to eliminate possible inconsistencies, through reading the abstracts. Those ones that were not related with the topic were discarded, reaching a final sample of 236 articles. The starting point of the period considered was set in 1991, when a first article including the search terms was published in a journal indexed in WoS. However, after double-checking, it was noted that it wasn't until 2009 when the first article relevant to the scope of this research was identified.

In order to analyse the scientific productivity, it was evaluated the number of publications; the distribution of articles by country, institution, journal, and author; and the most cited articles in the scientific literature, considering both the absolute and relative numbers of citations. Additionally, relational techniques were used to identify the structure of the research topic through science mapping analysis using the VOS Viewer software [80], which permits visualisation of various networks resulting from the analysis of co-words and co-citations of documents, authors, and journals. Finally, a bibliographic coupling analysis was carried out to identify the hottest research trends.

## 4 Results

### 4.1 Number of publications

The evolution of the academic literature on digitalization in the luxury sector can be divided into several key phases (RQ1), marked by an increasing interest in the topic in the most recent years. While the number of publications was initially low, the last years have witnessed a significant rise in the number of articles exploring different aspects related with the digitalization of the luxury industry. It is possible to identify three stages in the development of the academic literature on this topic: *seminal works* (2009–2015); *research settlement* (2016–2019); and *take-off* (2020 onward). These phases reflect the growing relevance of digital transformation and its impact on business strategies in the luxury sector.

In the first stage, named *seminal works* and running from 2009 to 2015, an incipient interest in the subject emerged consisting of the study of adaptations to the digital environment that luxury brands were beginning to implement. From the start of the digital revolution in the 1990s with the creation of the World Wide Web, nearly 25 years passed before the scientific community began to develop real interest about the digitalization of the luxury sector. Okonkwo [42] published the first article on this topic, analysing how luxury brands were facing the challenges posed by the development of the internet and digital environments. Specifically, this article highlights seven main challenges that luxury brands face in maintaining their exclusivity online. It is identified that many brands still have work to do in implementing standard e-business procedures, ranging from digital marketing to e-commerce.

One explanation for the early lack of interest in the subject was the resistance shown by luxury companies to any kind of change, as the strengths of the sector often include a basis in tradition and history. It is therefore not surprising that resistance to change would be characteristic of this industry. However, a shift in that attitude began to be reflected during this early period as international luxury brands were compelled to enter the digital age, creating their first websites and implementing new technological tools. Implementation proved very slow, explaining the scarce presence of this topic in the academic literature in the initial years under study. With these first publications, certain concerns become evident about two phenomena that would eventually attract greater attention: on the one hand, the effect of social networks on luxury brands [81], and on the other, the success via digitalization experienced by those luxury companies that took risks and began their transformation [60].

In the second stage (*research settlement*), from 2016 to 2019, there was moderate growth in the number of publications dealing with the subject of this study. This includes studies referring to the adoption of new digital tools for the sale and provision of services, as well as those examining the brand image projected through websites. Research on online sales and advertisements can also be highlighted [82]. While in the first stage academic interest was focused more on the birth of social media, during the second stage, that topic was somewhat abandoned in order to place greater emphasis on the use of digital resources.

During the third stage (*take-off*), the birth of new technologies or new internet-based services became an attractive topic for the scientific community, with focus on aspects such as the momentum of blockchain [39,83], 3D technology [84], and new luxury services available via the internet [85]. From 2020 onward, exponential growth showed that interest in the impact of social media on the luxury sector had once again gained importance within the scientific community, complemented by ongoing interest in the topics covered in the second stage. The continuous development of ICTs, the growing need to adopt them, the impact of Covid-19, and the transition to an online marketplace were the main factors that motivated an increased use of digital tools by luxury brands. Additionally, there was an increase in online sales [86], as well as the internationalisation of markets leading to globalisation of the sector and, therefore, an increase in the use of e-commerce [87]. Lastly, the projection of luxury brands through social networks has generated considerable interest [31,38,88] as well as the phenomenon of “influencers”, who have become the new ambassadors of luxury [89].

### 4.2 Authors by country and institution

One of the purposes of this research is to establish the intellectual structure of the subject. To this end, the leading academic schools and theoretical frameworks that have shaped the research on digitalization and digital transformation in the luxury sector have been identified. This approach provides a comprehensive understanding of the dominant academic perspectives and partially addresses RQ3.

**Fig. 2:**
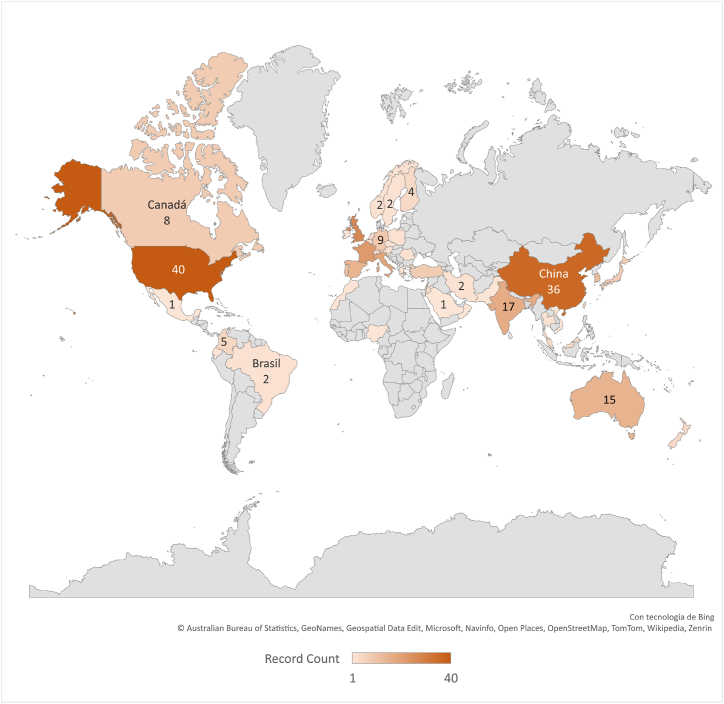
Number of WoS publications on digitalization in the luxury sector by country.

**Source:** Own elaboration.

As shown in Fig. 2, the largest numbers of publications come from countries that hold a strong relationship with the luxury sector, whether as producers or as consumers. The ten countries with the greatest volume of academic publications include European countries (the United Kingdom, France, Italy, and Spain) as well as the U.S., China, and India.

The largest luxury producers can still be found in Europe, where the main operational bases of the sector's brands and groups are located [90]. Examples include France (LVMH and Kering), Italy (Luxottica Group and Prada Group), the United Kingdom (Burberry and Jenny Packham), and Spain (Puig and Sociedad Textil Lonia). On the other hand, countries such as the U.S., China, and India stand out as major consumers. These are nations with the largest numbers of billionaires [91]– a market with great potential – leading to the considerable development of luxury brands such as Coach (U.S.), Titan Company Limited (India), and Eastern Gold Jade Co (China). While the North American market offers luxury brands across all sectors, luxury companies in China and India have tended to concentrate more on the precious metal and gemstone industries.

Next, we turn to those institutions responsible for the greatest numbers of publications. Hong Kong Polytechnnic University, located in China (which ranks second as a country) holds the first position. This university offers bachelor's and master's degrees specialised in the management of luxury brands and hotels, and its research focuses on diverse aspects from the study of social networks [92] to the use of blockchain [93]. Its researchers have also published several articles on luxury hotels, as this institution remains very focused on hospitality studies – even hosting a five-star hotel, where students are allowed to train [94].

The University of Florence is the second institution in terms of publications, which are mainly centred on e-commerce [87], with social networks also attracting interest [55]. The work of Ko et al. [95], co-authored by Professor Aiello Gaetano from the University of Florence, can be remarked amongst its scientific production.

University of South Carolina (U.S.) is placed in the third position. The topics by its academics range from luxury e-commerce to brand perception in the digital sphere. New York University, in turn, offers an MBA specialising in luxury and covering the fashion, retail, and marketing sectors. This breadth of research areas is reflected in the diversity if its academic publications, ranging from social media visibility [86] to online pricing strategy [35].

Yonsei University is also among the most prolific ones in this field, including the works of the leading author on the subject – Eunju Ko –, who works in the Department of Clothing and Textiles. The five articles from this institution were written by Ko in co-authorship with other researchers, analysing the implementation of new technological tools in luxury companies. More recent articles from this institution have considered the impact of Covid-19 on the sector [44].

The University of Manchester has published four articles, focused on the study of marketing. Two well-defined research lines can be distinguished: the study of the “influencer” phenomenon, carried out by Shuang Zhou; and study of the luxury market in China.

Finally, many other institutions registered a relatively low number of publications on this subject. This clearly reflects the fact that, although there is a growing interest in the subject, attention remains very dispersed, and there is no evident concentration of working research groups.

### 4.3 Publications by author

Also regarding RQ3, from the perspective of the intellectual framework, the 236 articles analysed in this paper were written by 567 authors. It is significant that the largest proportion of these authors (93.12 %) have written only one article on digitalization in the luxury sector, while a 4.94 % of the authors have published two papers, and 1.94 % are authors of three publications.

From another point of view, the analysis of the number of authors per article shows that 20.8 % of these works were written by a single author, while the highest percentage of publications (30.1 %) were by two authors, followed by research carried out by three authors (27.1 %). The majority of the researchers with the highest numbers of publications have participated in works with more than one author, with the exceptions of Jung-Hwan, Varsha Jain and Yuri Lee.

The most prolific authors on the subject, Eunju Ko and Yuri Lee, are of South Korean origin. Nonetheless, that country is ranked ninth in the number of publications. In contrast, while the UK, France, Australia, and Portugal all produce a considerable volume of publications (31 %), none of their authors have published more than three articles. Considering that 49 of the 236 papers selected were written by a single author, while 528 of 567 researchers published only one paper in this field, a relative dispersion of the is confirmed – i.e., there is no real concentration of papers by research teams in the most prolific countries and institutions. Thus, the empirical evidence indicates that this field of research is still in a phase of growth; greater numbers of publications and the consolidation of research teams would be required for the field to reach fully maturity [96].

As detailed above, certain countries (such as South Korea, China, and Italy) host a significant number of productive researchers in this topic, allowing them to reach significant positions in terms of productivity. Eunju Ko stands out with five articles written in collaboration with other authors, including members of her department. She repeats co-authorship in only two of these papers (with Kim S.J., these being the only publications by the latter). Eunju Ko is attached to Yonsei University, within a department specialised in the luxury sector with a marketing orientation (Fashion Marketing, Luxury Brand Management, and Social Media Marketing). She has published 89 articles available in the WoS database, most of them in the area of Business Management, covering topics related to both consumer behaviour and digital tools in luxury companies.

The second most prominent author from South Korea is Yuri Lee, affiliated with the University of Seoul, whose research focuses on fashion merchandising and retailing. Her work on the luxury sector focuses on consumer perception. Two of the three articles included in the selection published by this author are co-authored by Kim, H., and the other one by Jung, Y.J. and Choi Y.J.

Another set of prolific authors work at Chinese universities. Among them, Rob Law (affiliated with the University of Macau) can be considered the most outstanding, having published four articles on this topic. His field of research is focused on digital transformation applied to tourism within the hotel sector. This author collaborated with other researchers, especially from his own university. China is also represented by Shubim Yu (affiliated with Peking University), whose work belongs to the fields of digital communication, luxury marketing, and marketing communication. Since 2021, this researcher has been part of the BI Norwegian Business School.

Finally, several authors are working at Italian universities, including Simone Guercini from the University of Florence, whose research have focused on marketing and business, mainly e-commerce.

### 4.4 Most cited articles

Dealing with RQ2, the main documents on the topic were examined. The most cited article in absolute terms analyses the problem of determining the origin of physical objects to verify the authenticity of luxury goods [39]. This study proposes the use of ontology applied to the blockchain as a way of verifying the origin of a luxury product in an increasingly international society with complex supply chains. Meanwhile, the most cited article in relative terms analyses chatbot service through digital platforms and customer satisfaction of luxury brands [30].

From the analysis of the most cited articles, two main lines of research can be determined: one focused on the use of digital tools, and the other one on the use of social networks. On the topic of digital tools, the article by Kim and Laskowski [39] has been the most cited one in absolute terms. Choi [93] proposes the use of blockchain technology to verify the origin of diamonds distributed through complex supply chains. Alongside blockchain, research has examined technologies such as chatbots, which allow for more personalised and exclusive customer service [30]. The scientific community has also studied the use of big data and its applicability in specific cases, both in relation to luxury hotels [97] and to measure the impact of social media marketing [98].

The research stream focused on the use of social networks has produced diverse works. Liu et al. [98] analyse the impact of social media marketing. Other studies are more specialised, such as Phan et al. [60], who analyse Burberry's strong positioning in the luxury sector thanks to the integral digitalization of its marketing strategy. This brand was a pioneer in adapting to the digital environment, using its social networks and 3D technology, as well as virtual fashion shows and holograms. All of this made the company one of the most ‘followed’ in social media, prompting a significant increase in its sales. Among the most cited articles, we also find relatively general works that study brand perception through opinions expressed on Twitter [99] – somewhat similar to the aforementioned application of big data technology in the measurement of marketing's impact on social networks [98]. Midway between digital tools and social media is the first published article on digitalization in the luxury sector, by Okonkwo [42], which looks at the problems of digitalization in luxury companies and their positioning through the internet.

Finally, two articles focus on marketing and brand equity in a more general way. Choi et al. [100] analyses any sort of digital communication by luxury companies, including social media, while Ko et al. [95] classify eleven selected papers from the 2015 Global Fashion Marketing Conference at the University of Florence. The aim of the latter research is to delve into luxury brand strategy and customer experience, with four different rankings: social media and digital marketing, value creation, customer experience, and retailing. This classification serves as an introduction to a special issue that aims to increase knowledge in the management of this category of brands.

### 4.5 Visual maps of citations, sources, and authors

**Fig. 3:**
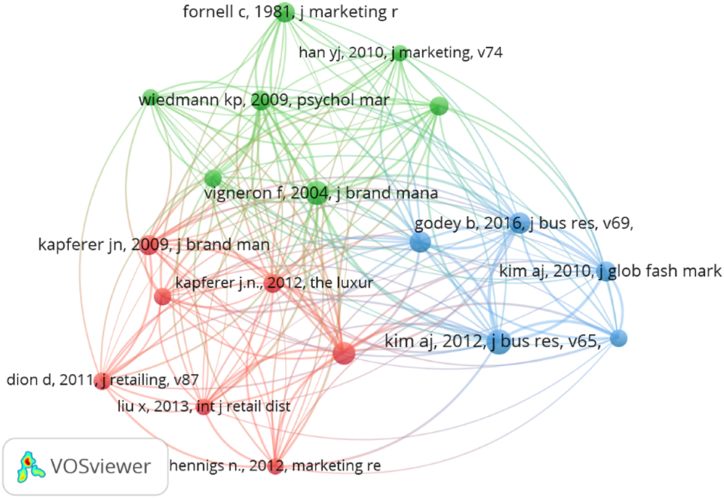
Document co-citation network in the field of digitalization in the luxury sector.

**Source:** Own elaboration.

To complete the answer to RQ2, co-citation analysis was used to enable the identification of the most influential works in this field of research, as well as their interrelationships [101]. Co-citation happens when an article simultaneously cites two or more different documents, authors, or sources, suggesting a certain degree of similarity among the elements cited [102]. A total of 12,179 cited references were found in the selected articles, 19 of which reached a threshold of 14 citations. Each node (Fig. 3) represents a reference, and its size indicates the number of citations obtained. A link between two nodes reveals a co-citation relationship. The thicker the line linking two nodes, the greater the strength of their relationship. Finally, nodes are understood to belong to different clusters depending on the degree of similarity between them.

The map below (Fig. 3) shows the existence of three clusters. The red one includes seven papers that are mainly related to the business management of luxury brands. Due to the similarity in the subject of these papers, high levels of representativeness and uniformity are observed in this cluster. Within the business management of luxury brands, the main topic of the whole cluster, two different approaches can be found: works focused on marketing strategy [103] and studies oriented to the adoption of digital tools [42] and online sales [104]. It is noteworthy that the works in this cluster were published in journals dealing with Business and Management.

In the blue cluster gathers different papers related with the use of social media. Godey et al. [105] and Kim and Ko [106] focus on the marketing aspect. In a different paper, Kim and Ko [107] approach this analysis from a communications perspective, while Phan et al. [60] analyse social networks as a digital tool. Tynan et al. [108] study both the creation of luxury brand value through customer interaction via company websites and the need to monitor the communication channels of these companies. Although they do not mention social networks directly, the relationship is implicit. Given the similarity of the works included in this second cluster, all of them were published in the same two scientific journals: the *Journal of Business Research* and the *Journal of Global Fashion Marketing*.

The green cluster collects works on the differentiating aspects of a luxury brand. Examples include the analysis carried out by Han et al. [109] on the logo of a brand, and the study by Wiedmann et al. [19] on the motives for consumption of luxury brands. Vigneron and Johnson [110] highlight the differentiating elements between a luxury company and other companies. Fionda and More [111] identify the critical dimensions to maintain success in a luxury company. The cluster also includes one publication by Fornell and Larcker [112] that is fundamentally statistical in nature, focusing on structural equations – a technique frequently used in the study of the digitalization of the luxury sector.

**Fig. 4:**
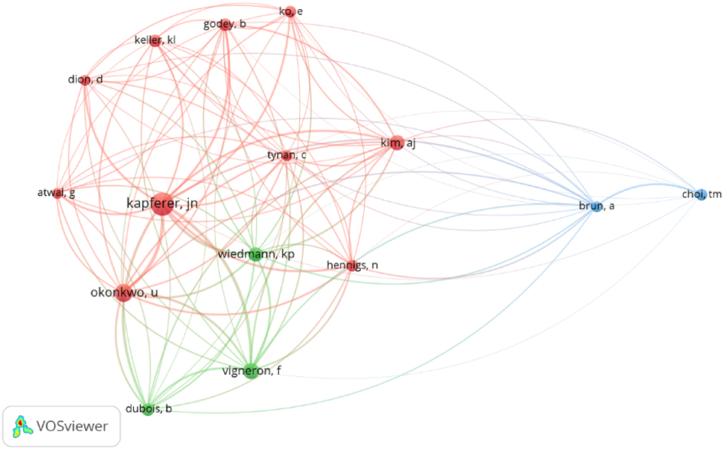
Co-citation network of authors in digitalization in the luxury sector.

**Source:** Own elaboration.

Fig. 4 displays the results of the analysis of citations by author. The papers studied cite works written by 8,995 authors, out of which 16 were cited a minimum of 26 times. However, this body of authors included a data platform known as *Statista*. As the aim of the present analysis is to identify the main authors grouped into clusters, the *Statista* reference was discarded, leaving a total of 15 authors.

The red cluster includes authors whose work is primarily concerned with brand strategy. Okonkwo [42] examines the role of luxury brands in the digital context. Jean-Noel Kapferer is distinguished for his comprehensive understanding of consumer behaviour, brand management, and strategic marketing within the luxury industry. The green cluster includes researchers who have focused most of their work on the luxury and marketing sectors. It should be noted that both Alex Jiyoung Kim and Eunju Ko, whose works are among the most cited, are included in this group. Finally, there are only two authors in the blue cluster: Alessandro Brun and Tsan-Ming Choi, whose main contributions in the field are limited to supply chains and digital tools, reflecting the importance of both in terms of the efficiency of the luxury sector.

A co-citation analysis of scientific journals was carried out in order to complete the answer to RQ3, making it possible to determine the similarity between journals and the area of research [61,113], allowing us to identify the academic schools and theoretical approaches. In the selection of 236 documents, a total of 5,429 journals were identified, 20 of which reached a threshold of 80 citations.

**Fig. 5:**
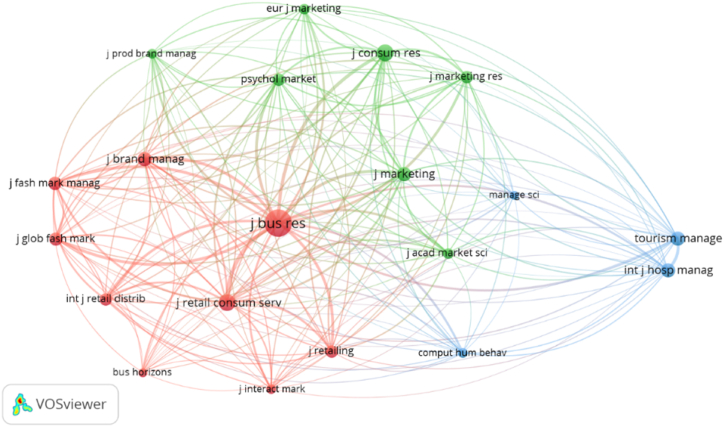
Co-occurrence network of journals in the field of digitalization in the luxury sector.

**Source:** Own elaboration.

References to digitalization in the luxury sector can be classified into three main groups of publications, differentiated by colour (Fig. 5).

Two groups – green and red – exhibit a certain level of similarity, as both contain publications with a general marketing focus. However, the red cluster also contains generalist publications on management, such as *Business Horizons* or the *Journal of Business Research*; publications related to the fashion sector, such as the *Journal of Fashion Marketing and Management* and the *Journal of Global Fashion Marketing*; and others journals related to retailing, such as the *Journal of Retailing and Consumer Services* and the *Journal of Retailing*. The red cluster gathers six of those the journals publishing the majority of the articles on digitalization in the luxury sector. The green cluster is dominated by journals focusing on marketing and consumer behaviour, such as the *European Journal of Marketing* and the *Journal of Marketing Research*. A third cluster, the blue one, comprises publications linked to the tourism sector as well as those related to ICTs: the *International Journal of Hospitality Management*, *Tourism Management*, *Computers in Human Behavior,* and *Management Science*.

As a result, a relationship is observed between the most cited journals and the research areas that constitute the foundations of digitalization in the luxury sector, namely: business management, supply chain analysis, marketing, tourism, and information systems.

### 4.6 Keyword co-occurrence

**Fig. 6:**
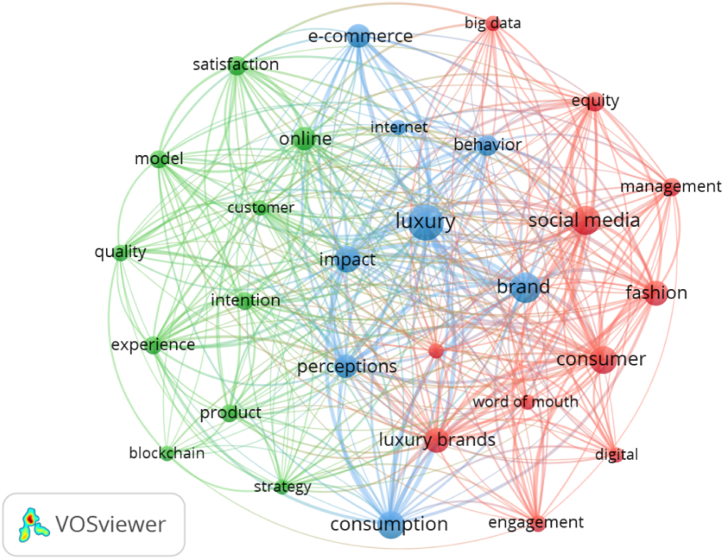
Co-occurrence network of keywords in digitalization in the luxury sector.

**Source:** Own elaboration.

A co-word analysis was performed to deal with RQ4, uncovering the most prominent research themes [114]. In the present study, 1,314 keywords were analysed, out of which 29 had a minimum of 12 occurrences (Fig. 6).

The classification of keywords into three clusters reveals the existence of three main research approaches to digitalization in the luxury sector. The blue cluster is mainly related to luxury, the impact of digitalization, and potential consumers (“luxury”, “Brand”, “impact”, “perception”, “behaviour”). The green cluster is particularly concerned with the design of experiences and product, just as interactions with target groups (“customer”, “quality”, “product”) as well as with business management and the use of digital tools (“blockchain”, “strategy”). The red cluster, in turn, is associated with marketing, communications, and value creation (“social media”, “word of mouth”) as well as the analytical tools used in social networks (“big data”, “digital”).

It is noteworthy to state that the term "product" initially referred to "luxury goods" in early studies, but over time it has evolved to encompass a new category of goods, reflecting a new trend in the luxury market. Products made with advanced technology or luxury digital products have become significant draws in the industry. Non-Fungible Tokens (NFTs) are particularly outstanding due to their diverse functionalities, offering attributes such as indivisibility, traceability, and verifiability. These NFTs can range from unique fashion items to simple certificates of authenticity for various products [115]. Additionally, devices like RFID (radio-frequency identification) technology are employed to verify product authenticity [116]. The rise of luxury digital products is also affecting the traditional market, as can be seen in the competition between new smartwatches and traditional luxury watches [117]. The introduction of these products into the markets has resulted in limited availability, creating a sense of scarcity and prompting status-conscious consumers to pursue such purchases.

Regarding products made with advanced technologies, three key aspects should be highlighted: First, despite studies suggesting a decline in brand perception for companies using AI in product design, this is not especially true for products whose primary value lies in their utility, such as automation in the automotive industry [118]. Second, AI has emerged as a leading tool for the online personalization of products [119]. Lastly, there is a focus on the use of 3D printing to enhance the luxurious appeal of products [120].

### 4.7 Recent research trends

A bibliographic coupling analysis was conducted to deal with RQ5. Bibliographic coupling is considered one of the most useful bibliometric techniques in order to establish the hottest topics related with a research field and anticipate the most probable future research agenda in this field [121]. Coupling takes place when two papers reference the same document, revealing a link between them, as they share the same empirical or theoretical foundations [61]. Therefore, bibliographic coupling complements the results of co-word analysis, delivering a deeper understanding of the most recent literature, becoming a popular tool amongst the academic community [122,123].

**Fig. 7:**
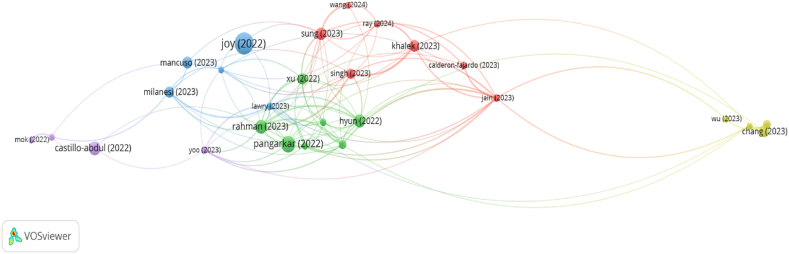
Bibliometric coupling analysis.

**Source:** Own elaboration

The identification of the trendiest research topics dealing with the digital transformation of the luxury industry has been carried out over 142 articles belonging to this research stream, published between 1/1/2022 and 30/6/2024, following the same search and selection strategy used in previous sections to carry out the productivity and mapping analyses. Amongst these papers, Fig. 7 represents those ones (31) which have been cited at least 6 times and have network ties with, at least, another article. As can be seen, these papers are gathered in five clusters, which identify the most promising research lines regarding the digital transformation of the luxury industry in the immediate future.

The green cluster is the most populated one, including seven different papers, which also totalize the highest number of citations (120). The articles grouped in this cluster mainly deal with the achievement of customer engagement in the digital luxury industry, usually trying to use ICTs in order to empower the brand image of a company/house. Therefore, both social media and the webs of luxury companies hold a core position in this cluster. The majority of these papers have been published in journals strongly related with marketing and, specifically, retail, though one of them is associated with hospitality management. The methodologies used are diverse, but there is an important presence of experimental approaches, together with the analysis of the content published by luxury companies in their digital channels.

The study of Pangarkar et al. [124] is focused on the exploitation of *phygital* capabilities of luxury firms, profiting from both online and traditional retail, to increase the engagement of their customers, using qualitative techniques to analyse the knowledge of 18 experts of this industry. Combining an exploratory qualitative approach with a PLS-SEM quantitative study, Rahman et al. [125] analyse the relationship between AI powered customer assistance, customer engagement and customer shopping experience, finding out the importance of digital multisensory cues. Hoang et al. [126] conducted a hybrid thematic analysis of the content of 96 YouTube videos from luxury companies, making it possible to provide some recommendations on the use of technological tools to increase customer engagement in a digitally transformed environment after the pandemic. The improvement of customer-based brand equity is the leitmotiv of Hyun et al. [127], who carried out a survey amongst 422 American customers who had previously experienced with luxury firms’ webs, finding out that emotional appeal, web design and customer service are the most critical items.

There is a generic concern about the impact of digitalization on the customer perception of luxury goods. Xu and Mehta [118] carried out an experiment amongst MBA students, finding out that AI design has a negative impact in the perception of the emotional value of a luxury brand, but may have a positive influence on the functional value perceived. Comparing the impacts of luxury brands in social media with other companies through an experimental design, Kemper et al. [128] found out that the contents delivered by luxury firms are more frequently shared by social media users when they are associated with material rather than experiential issues, and amongst consumers with a high sense of entitlement. Finally, Michael and Fuste-Forne [129] use a visual analysis methodology with the aim of identifying the features which are more likely to catch the attention of the customers related with the gastronomy offered by the restaurants of luxury Emirates’ hotels.

The red cluster also includes seven papers, most of which are literary reviews, using different bibliometric techniques, revealing at the same time a raising concern of the academic community about this topic and its relative immaturity. Along with them, three articles deal with the mechanisms that guide the perceptions of the customers. The journals that have published these articles deal mainly with consumer studies and luxury hospitality management, revealing again the relevance of this specific part of the industry.

Amongst the literary reviews, three different bibliometric analyses were performed dealing with brand personality [130], luxury hospitality industry [131] and shared consumption [132], while another study deals with the online consumer behaviour using the theory, context, characteristics and methodology (TCCM) framework [133]. More specifically related with consumer behaviour, Sung et al. [134] analysed using a quantitative approach (PLS-SEM), based on a survey carried out amongst metaverse participants, the factors that push consumers to purchase NFTs associated with the luxury industry. Ray et al. [135] compare the enablers and barriers revealed by the users of luxury hotels through a mixed qualitative (structured interviews) and quantitative (survey) methodology with the results of a content analysis of the web of these companies, finding a relevant mismatch between the demands of the customers and the communication of the firms. Within the restauration industry, Wang and Papastathopoulos [136] found out, using a quantitative approach (survey), that trust in technology is critical in the acceptance by the customers of luxury, fine dining, casual and quick restaurants of AI-based service robots, being this relationship stronger amongst males.

The yellow cluster is focused on the luxury hospitality industry, and the information is gathered through an analysis of the reviews of the customers after their experience with these companies. Therefore, the analytical techniques used are mainly quantitative, though they range from a simpler analysis of the features preferred by the customers to a more sophisticated sentiment analysis. While two of these articles are published in technological journals, the remaining two ones directly targeted hospitality management publications.

Chang et al. [137] propose a heuristic model for sentiment analysis on luxury hotel reviews to analyse and explore marketing insights from attitudes and emotions expressed in reviews, validating this model for further evaluations. Sentiment analysis based on the reviews of the customers is also used by Chen et al. [138] in order to evaluate the relationship between the content of these reviews and real features regarding luxury hotels. Using thematic salience valence analysis and lexical salience valence analysis, Kajla et al. [139] identified staff, location, food, hygiene and rooms as the preferred hotel attributes when choosing a luxury accommodation along COVID-19 crisis. Lastly, Wu et al. [37] use machine learning to analyse 317,518 online reviews of luxury hotels to identify possible innovations and improvement opportunities, using an open innovation and value cocreation approach.

The blue cluster gathers five papers, including the most cited one of the entire sample, mainly concerned with value creation strategies and practices in the luxury fashion industry through the use of different digital technologies, including NFTs, the metaverse or gamification experiences [115]. As this topic can be boarded from different perspectives, these articles have been published in journals related with the luxury industry, strategic management or e-commerce. The collection of methodologies is also wide, ranging from theoretical reflections or the design of conceptual models to experimental approaches.

Based on different examples from the luxury fashion industry, Joy et al. [115] reflect on its probable evolution, wondering if in a near future new consumers will be more interested in digital luxury products, such as NFTs, than in traditional luxury goods, and how can the industry manage the coexistence of both, digital and physical worlds. The answer, is going *phygital*, trying to profit from the best of both worlds [140,141]. Lawry [140] deals with the generation of *phygital* luxury experiences through mobiles service activities, designing a conceptual framework which reveals the main status and hedonic drivers for the different kinds of service activities. Mancuso et al. [141], in turn, analyse the evolution of the business models of four companies in order to profit from the opportunities generated by the metaverse, considering either a complete digitalization or, especially, the use of a *phygital* approach. Through 11 interviews with luxury market experts and consumers, Alexander and Bellandi [41] reflect on the potential of NFTs to create value and guide the strategies of luxury companies. Finally, Milanesi et al. [55] investigate the potential of gamification as a digital marketing tool to deliver a digital luxury experience, through the analysis of a specific gamification experience of a luxury firm.

A fifth and last trend, developed in the papers included in the purple cluster, analyses the role played by the digitalization of the luxury industry in achieving sustainable goals or, at least, improving its perception of sustainability. Dealing with perceptions, communication in general, and particularly social media are positioned in the core of this topic, which is developed by papers mainly published in marketing and communication journals.

Using a quantitative methodology (ANOVA analysis), Castillo-Abdul et al. [142] analyse the kind of Instagram contents which generate more impact in the followers of luxury brands, as they use branded content related to social responsibility. The authors conclude that branded content contributes positively to brand reputation. Also working with Instagram, Yoo [143] compare successful contents of luxury and fast fashion brands, through a quantitative analysis of the photos posted by the companies, in terms of the use of a brand name, brand logo, text and hashtag, finding that brands, logos and text embedded in images generates positive reactions in luxury fashion brands compared with fast fashion ones. The evolution of the communication of sustainability commitments of luxury brands is studied by Mok et al. [144], using different content analysis techniques to study, through a Tripple Bottom Line perspective, 32 years of newspaper articles published by Women's Wear Daily (frequently referred to as the bible of fashion). Finally, Akrout and Guercini [145] contributed a guest editorial article that reflects on marketing and management research concerning the sustainability of fashion and luxury companies, summarizing the various articles included in a special issue dedicated to this topic.

## 5 Conclusions and future research lines

### 5.1 Conclusions

In the context of the so called fourth industrial revolution, characterized by the massive and strategic use of ICTs in any area of our lives, there is a need to analyse the digital transformation of the economy, considering particularly the specific needs and features of the different sectors.

The digital transformation of the luxury industry has relevant particularities that make it especially complex. Luxury brands need to balance digital innovation with their core values of exclusivity and heritage, ensuring that their brand identity remains intact. The use of ICTs in the luxury industry presents both opportunities and challenges. A strategic approach, which is embedded in digital transformation and not necessarily in digitalization, should grant that technology is aligned with the brand's luxury ethos and enhances consumer engagement. By doing so, luxury brands can not only navigate the complexities of the digital age, but also thrive into it, securing a competitive edge in the ever-evolving market landscape. This requires a careful selection and use of digital channels and tools that preserve and elevate the brand's luxury status.

The objective of this study has been to disentangle the knowledge structure of the academic literature dealing with the digital transformation and digitalization of the luxury industry through a bibliometric analysis.

In the examination of the historical evolution of the academic literature (RQ1), three different phases have been identified, characterized by a growing interest in the topic and an increase in the implementation of digital tools. Regarding the key documents in this research stream (RQ2), two major theoretical streams have emerged: social media and specific digital tools. The analysis of the leading academic schools and theoretical approaches (RQ3) reveals that this research stream lays in the middle of different disciplines, such as technology, hospitality management, marketing, business administration … The most prolific countries in terms of publications are those with significant consumption and/or production of luxury goods. The absence of large, dedicated research teams reveals a relative immaturity of this research field, anticipating a promising development.

The techniques dealing with RQ4 have revealed that the three core themes in the past literature dealing with this topic are the impact of digitalization, the design of experiences and products, and marketing strategies. Regarding the analysis of the most probable future research agenda (RQ5), It could expect that the literature would focus on the use of digital tools to enhance customer engagement; exploring the tourism sector, with a particular emphasis on review-based feedback; investigating value creation practices through innovative products or promotional strategies; and advancing sustainability efforts, particularly through digitalization as a tool for achieving and communicating sustainability goals to customers.

In addition to qualitative and quantitative approaches, experimental methods can be considered an interesting methodological option to create fresh research on digitalization in the luxury sector. Experiments allow the establishment of causal relationships and precise measurement of the impact of digital interventions. Viglia and Dolnicar [146] highlight the external validity that well-designed experiments provide, which is essential to apply its findings in real-world contexts. Chan and Northey [147] use online experimental designs to test how product presentation influences consumer decisions, demonstrating the importance of variable control and randomization to ensure robust results.

Amaral and Loken [148] employ an experimental approach by manipulating social conditions to analyse consumer perceptions of counterfeit products, emphasizing the role of control groups and comparative conditions. Similarly, Kluge and Fassnacht [149] conduct controlled experiments to assess the impact of digital accessibility on perceptions of exclusivity, ensuring internal validity through randomization and controlled experimental conditions. These methodological approaches underline the importance of rigorous experimental designs in future research on digitalization.

Academic research highlights the critical role of social media and e-commerce in expanding the reach of luxury brands, facilitating personalised and interactive consumer experiences, and fostering deeper connections with their audiences. The use of big data and AI enhances these efforts by providing valuable insights into consumer preferences and behaviours.

Luxury brand websites are essential for replicating online the exclusive in-store experience, acting as key sales tools that cater to diverse consumer needs—whether for status-driven, intelligent, ostentatious, or discreet purchases. For those who value discretion and intelligent purchase, online platforms offer the ideal level of anonymity and information.

Maintaining the essence of the shopping experience as an integral part of the product requires significant investment and effort, including the implementation of adequate pricing strategies, the adaptation to the needs of new generations, and the measurement of consumer behaviour through profiling. Many recent studies are embracing the term *phygitalization*, referring to experiences that blend real-world and digital attributes in the design of a luxury experience. The phygital experience, exemplified by innovations such as virtual fitting rooms in physical stores and chatbots in digital environments, is increasing its presence in the luxury landscape.

Review platforms, especially in the hospitality industry, have become increasingly influential. It is critical for companies to actively engage with these platforms, giving feedback, addressing concerns, and using reviews to drive improvements. Review platforms also impact product consumption, as customers discuss the most and the least valued attributes.

Social media remains a core concern for luxury brands, and a productive research area. Influencers, as modern brand ambassadors, promote high-end lifestyles, stimulating consumer desire for luxury products. Through social media, brands project their image, build narratives, and engage with consumers. Media content platforms allow brands to produce short stories, broadcast fashion shows and understand consumer preferences through viewer engagement.

Emerging technologies such as AI, blockchain, big data, and NFTs could significantly impact the luxury industry. Practical applications include the systematic analysis of customer reviews through machine learning; product personalization using AI; and the standardisation of consumer attention. Additionally, measuring the effectiveness of social media marketing and delivering personalised messages allow luxury companies to build a unique consumer experience.

Digital transformation also enhances sustainability by enabling transparent communication of company policies, tracking supply chains, optimising resources, and adopting sustainable materials, all of which positively influence brand loyalty.

The academic literature analysed in this paper unanimously recognises the importance of digital transformation for the luxury sector, showing that some initiatives are not just strategic choices, but essential actions required to maintain their level of competitiveness in the market. However, our findings reveal that a complete digital transformation has not yet taken place in the whole luxury sector but just in a bunch of cases, as most companies have only digitalized some of their activities, implementing specific ICTs. Consequently, most of the academic literature mainly deals with these digitalization practices rather than studying the digital transformation of companies and/or subsectors.

This situation leads us to formulate two additional questions that can be boarded by future academic research. Firstly, can classic luxury companies survive by merely incorporating digital tools, without undergoing a true digital transformation? And secondly, is it possible to develop successfully a new luxury brand or product within an organization that has not been entirely digitally transformed?

This study fills a gap in the academic literature, as no prior bibliometric analysis has comprehensively covered the adaptation of the luxury industry to digitalization and digital transformation, including the full spectrum of digital tools and sectors. It offers valuable insights about the integration of digital technologies in the luxury sector, highlighting the key managerial implications. The findings emphasize that digital transformation is essential for maintaining competitiveness. Managers must recognize that digital tools and platforms are not merely complementary to traditional luxury strategies, but are essential for engaging a modern, tech-savvy consumer base.

According with the evolution of the environment, and as a result of this analysis, we consider that luxury companies will only be viable in a very immediate future if they undertake a complete digital transformation. Therefore, this paper can be a valuable source of academic information for companies and all sectors of the luxury industry, which can benefit from the wealth of knowledge provided by the academic literature as they embark on their journey to full digital transformation.

### 5.2 Limitations and future perspectives

Among the main limitations of the present study, we can remark the selection of papers for the analysis. Although the chosen Web of Science database brings together a majority of the most outstanding publications, a few relevant studies could have been excluded from it, thus limiting the quantity of papers selected. Nevertheless, we can consider that the sample of papers analysed in this study is sufficiently representative of the academic literature dealing with the topic. Additionally, as in any bibliometric analysis, the interpretation of the results achieved could be somewhat subjective.

The field of digitalization in the luxury sector presents numerous opportunities for further exploration, particularly as emerging technologies continue to reshape consumer behaviour and business practices. To advance the research in this domain, several critical directions should be considered.

First, longitudinal studies could provide valuable insights into the impact of digitalization over time. These studies could track how luxury brands evolve in response to digital transformation and consumer expectations. For instance, the long-term effects of digital marketing strategies on brand equity and customer loyalty remain underexplored.

Second, there is a need for qualitative research methods, such as case studies and in-depth interviews, that focus on the internal decision-making processes within luxury firms. These methods could uncover the challenges brands face in integrating digital technologies while maintaining their core values of exclusivity and heritage.

In terms of data, future research would benefit from designs that combine quantitative surveys with big data analytics. This approach would allow researchers to capture the breadth of consumer behaviour through large-scale data collection, while also providing deeper insights through qualitative analysis of consumer sentiment on digital platforms such as social media.

Moreover, the ethical dimensions of digitalization, such as data privacy and the potential commodification of exclusivity through digital platforms, deserve more attention. Understanding how luxury consumers perceive these changes is critical for both academic inquiry and practical application.

While significant progress has been made in studying digital transformation within the luxury sector, many existing studies focus narrowly on specific industries, such as fashion or social media marketing. But lacks depth in evaluating long-term impacts on brand identity and customer retention. Similarly, much of the academic literature relies on cross-sectional analyses, which, while useful, fail to capture the dynamic and evolving nature of digitalization**.**

There is also a gap in understanding the integration of advanced technologies like AI. Thus, future research should adopt a multidisciplinary approach, integrating perspectives from different fields such as sociology, technology studies, and business ethics to provide a more comprehensive view of how digital tools are reshaping the luxury industry.

One of the most significant gaps in the current literature on digitalization in the luxury sector is the absence of a comprehensive analysis that measures the full impact of digitalization on business profitability and viability. While numerous studies focus on the adoption of specific digital tools, such as social media marketing, e-commerce platforms, or AI-driven customer service, there is a lack of research that evaluates whether a full-scale digital transformation is beneficial—or even feasible—for luxury brands. This raises important questions about the optimal level of digitalization or digital transformation for maintaining brand exclusivity while enhancing operational efficiency.

The academic literature to date has largely focused on isolated digital tools without examining the broader implications of a holistic digital strategy. For example, while there is more than sufficient research on the adoption of digital marketing or blockchain for product authentication, it remains unclear whether these tools, when combined into a fully integrated digital framework, lead to sustainable business models. There is also no clear understanding of whether a complete digitalization of the luxury sector would erode the core values of craftsmanship, heritage, and exclusivity that luxury brands rely upon for their distinct market positioning.

Additionally, the cost of digital adaptation is rarely addressed in the literature. The financial investment required to implement digital solutions—ranging from AI-driven personalization to blockchain for supply chain transparency—is significant, yet there are few studies that attempt to measure the return on investment (ROI) or the long-term competitive advantages gained through these investments. Understanding the cost-benefit relationship of digital tools is essential for luxury brands to make informed decisions about which technologies to adopt and to what extent.

Moreover, the question of what constitutes the "optimal" digital strategy remains unanswered. It is unclear if there is a threshold where further digitalization ceases to provide additional benefits or even becomes detrimental to the brand's identity and customer base. Does the incremental implementation of digital tools (such as chatbots, virtual fitting rooms, or personalised marketing algorithms) lead to greater success than a rapid, full-scale digital overhaul?

Future studies must address these critical gaps by developing empirical models that quantify the impact of digitalization on business outcomes, including profitability, operational efficiency, and brand equity. Specifically, research should focus on the following areas:

Measuring the optimal level of digitalization: Researchers need to develop frameworks that assess how far luxury brands can digitalize without compromising their core values. This involves identifying the point at which further digitalization no longer yields proportional benefits in terms of efficiency or customer engagement.

Cost-benefit analysis of digital tools: Future studies should aim to quantify the financial costs associated with adopting digital technologies and compare these with the competitive advantages gained. This analysis should also consider the long-term sustainability of such investments.

Research should empirically test the improvements in operational efficiency attributed to specific digital tools. Studies could explore how the integration of AI or blockchain affects areas such as supply chain transparency, production times, or customer service responsiveness. Finally, future research should aim to identify which digital tools provide the greatest return on investment for luxury brands. This would involve a comparative analysis of technologies like AI, big data, blockchain, and virtual reality, determining which are most effective in enhancing brand performance without diluting exclusivity.

To address the current lack of a standardized approach to measuring the impact of digitalization in the luxury sector, this paper proposes the development of a Digital Maturity Index. Such an index would serve as a framework to assess the extent to which luxury companies have integrated digital technologies into their operations, marketing, and customer service. The Digital Maturity Index could evaluate multiple dimensions, including the degree of automation, the use of big data for consumer analytics, and the incorporation of AI-driven personalization. By quantifying the level of digital maturity across these axes, researchers and industry professionals would be able to better understand the relationship between digitalization and key business outcomes, such as profitability, brand loyalty, and operational efficiency. This index could serve as a benchmarking tool, allowing companies to compare their digital capabilities with competitors and identify areas for further improvement.

In addition to a general digital maturity index, this paper advocates for the creation of a Tool-Specific Digital Index that would assess the effectiveness of individual digital tools across various business functions. This index would be structured around key axes or dimensions, each corresponding to a specific digital tool—such as AI, blockchain, e-commerce platforms, and social media marketing. Each axis would measure the impact of the respective tool on efficiency gains, customer engagement, and competitive advantage. This granular approach would enable luxury brands to evaluate which tools are driving the most significant improvements and where additional investment might be warranted. Moreover, this index could provide valuable insights into which tools offer the greatest return on investment for specific luxury segments, thereby guiding more strategic decision-making in digital adoption.

## CRediT authorship contribution statement

**Francisco Sanz-Lopez:** Writing – review & editing, Visualization, Validation. **Rocío Gallego-Losada:** Writing – original draft, Validation, Supervision, Resources, Formal analysis, Conceptualization. **Antonio Montero-Navarro:** Writing – original draft, Validation, Supervision, Formal analysis. **Elisa García-Abajo:** Writing – review & editing, Visualization, Validation.

## Declarations

On behalf of all authors, the corresponding author states that there is no conflict of interest.

The authors have no relevant financial or non-financial interests to disclose.

No funding was received for conducting this study.

This research did not receive any specific grant from funding agencies in the public, commercial, or not-for-profit sectors.

## Data availability

Data will be made available on request.

## Declaration of generative AI and AI-assisted technologies in the writing process

During the preparation of this work the authors used deepl and ChatGPT to improve same expression in English. After using this tool, the authors reviewed and edited the content as needed and takes full responsibility for the content of the publication.

## Declaration of Competing Interest

The authors declare that they have no known competing financial interests or personal relationships that could have appeared to influence the work reported in this paper.

## Acronyms

(ICTs): Information and communication technologies
(AI): Artificial Intelligence
(DT): Digital Transformation
(WoS): Web of Science
(NFTs): Non-Fungible Tokens
(ROI): Return On Investment
